# Development of novel apoferritin formulations for antitumour benzothiazoles

**DOI:** 10.1002/cnr2.1155

**Published:** 2019-01-16

**Authors:** Alastair F. Breen, Geoffrey Wells, Lyudmila Turyanska, Tracey D. Bradshaw

**Affiliations:** ^1^ Centre for Biomolecular Sciences, School of Pharmacy University of Nottingham Nottingham UK; ^2^ School of Pharmacy University College London London UK; ^3^ School of Physics and Astronomy University of Nottingham Nottingham UK; ^4^ School of Chemistry University of Lincoln Lincoln UK

**Keywords:** anticancer activity, apoferritin, benzothiazole, drug delivery, transferrin receptor

## Abstract

**Background:**

The benzothiazole structure is important in medicinal chemistry, and 5‐fluoro‐2‐(3,4‐dimethoxyphenyl) benzothiazole (GW 610) is of particular interest as it shows outstanding anticancer activity in sensitive breast and colorectal carcinoma cell lines via generation of lethal DNA adducts in sensitive cancer cells. Despite promising activity, poor water solubility limits its applications. The apoferritin (AFt) protein cage has been proposed as a robust and biocompatible drug delivery vehicle.

**Aims:**

Here, we aim to enhance solubility of GW 610 by developing amino acid prodrug conjugates and utilizing the AFt capsule as drug delivery vessel.

**Methods and results:**

The potent experimental antitumour agent, GW 610, has been successfully encapsulated within AFt with more than 190 molecules per AFt cage. The AFt‐GW 610 complex exhibits dose‐dependent growth inhibition and is more potent than GW 610 alone in 5/7 cancer cell lines. To enhance both aqueous solubility and encapsulation efficiency, a series of amino acid esters of GW 608 prodrug were synthesized via *N,N′*‐dicyclohexylcarbodiimide ester coupling to produce molecules with different polarity. A dramatic increase in encapsulation efficiency was achieved, with more than 380 molecules of GW 608‐Lys molecules per AFt cage. Release studies show sustained release of the cargo over 12 hours at physiologically relevant pH. The AFt‐encapsulated amino acid modified GW 608 complexes are sequestered more rapidly and exhibit more potent anticancer activity than unencapsulated agent.

**Conclusion:**

These results indicate that AFt‐encapsulation of GW 610 prodrug provides a biocompatible delivery option for this potent, selective experimental antitumour agent and for amino acid‐modified GW 608. Of particular interest is the encapsulation efficiency and in vitro antitumour activity of AFt‐GW 608‐Lys, which warrants further preclinical evaluation.

## 1 INTRODUCTION

Advancement of novel pharmaceutical agents to the market is often thwarted by lack of selectivity and solubility in physiological solvents. Various nanoscale delivery vehicles have been considered to address these issues, such as liposomes, micelles, and protein capsules. Of particular interest is protein capsule apoferritin (AFt), which has been successfully used for development of biohybrid and self‐assembled materials,^1^, ^2^ for the encapsulation of proteins^3^ and small drug molecules.^4^, ^5^, ^6^, ^7^, ^8^ AFt consists of 24 polypeptide subunits assembled into a spherical protein cage with an internal cavity of 8 nm in diameter^9^; 0.3‐ to 0.4‐nm channels in the shell^10^ and pH‐dependent dissembly^9^ allow controlled release of cargo. The uniform size, biocompatibility, biodegradability, and non‐toxicity has led to AFt being identified as an ideal drug delivery vehicle.^11^ AFt is internalized into cells by transferrin receptor (TfR)‐mediated endocytosis; TfRs are upregulated and highly expressed on cell membranes in many cancers (including colon and breast cancinomas).^12^ AFt as a drug delivery vehicle can lead to enhanced delivery to cancer tissue, via exploitation of the enhanced permeability and retention (EPR) associated with the tumour micro‐environment,^13^ leading to increased intracellular drug concentrations within a tumour, improving the therapeutic efficacy and decreasing side effects.^14^

The benzothiazole structure is important in medicinal chemistry, as derivatives of this scaffold possess anticancer,^15^ antibacterial,^16^ antiviral,^17^ antimalarial,^18^ and antifungal^19^ activity. From this scaffold, a chemistry‐driven drug discovery project resulted in the development of a wide range of 2‐phenylbenzothiazoles with oxygenated substituents in the phenyl moiety. In a library of more than 35 close structural analogues, one compound 5‐fluoro‐2‐(3,4‐dimethoxyphenyl) benzothiazole (GW 610), showed outstanding potent and selective anticancer activity against, eg, colorectal and breast carcinoma models.^15^ GW 610 is a potent aryl hydrocarbon receptor (AhR) ligand, bioactivated via cytochrome P450 (CYP) 1A1 and 2W1 catalysis to electrophilic species that generate lethal DNA adducts in sensitive cancer cells.^20^, ^21^, ^22^

However, GW 610 is highly lipophilic and poorly water soluble; hence, drug delivery tools are needed to help solubilize and target GW 610. During biotransformation, GW 610 initially undergoes regiospecific demethylation to give 5‐fluoro‐2‐(4‐hydroxy‐3‐methoxyphenyl) benzothiazole (GW 608).^21^ The exposed 4′‐hydroxy group in GW 608 allows conjugation of substituents via an ester linkage to the benzothiazoles and could lead to enhanced aqueous solubility and stability, whilst retaining antitumour activity.

We report AFt encapsulation of GW 610, GW 608, and amino acid conjugates of GW 608 to address water solubility and selectivity issues associated with benzothiozoles for anticancer treatment. Amino acid conjugation enhances drug solubility and modifies the charge and polarity, thus allowing optimization of the properties of cargo molecules to achieve increased encapsulation efficiency and controlled sustained release. In this work, we consider nonpolar glycine (Gly), polar serine (Ser), positively charged lysine (Lys), and negatively charged aspartic acid (Asp) conjugated to GW 608. The in vitro antitumour activity of the AFt formulations was investigated against MDA‐MB‐468, MCF‐7 breast, IGROV‐1 ovarian, TK10 renal, KM‐12, HCC‐2998, and HCT 116 colorectal carcinoma (CRC) cell lines. Our study of novel benzothiazole formulations reveals exciting prospects for their development as anticancer agents.

## 2 RESULTS AND DISCUSSION

### 2.1 Synthesis and AFt encapsulation of amino acid conjugates of GW 608

**Figure 1:**
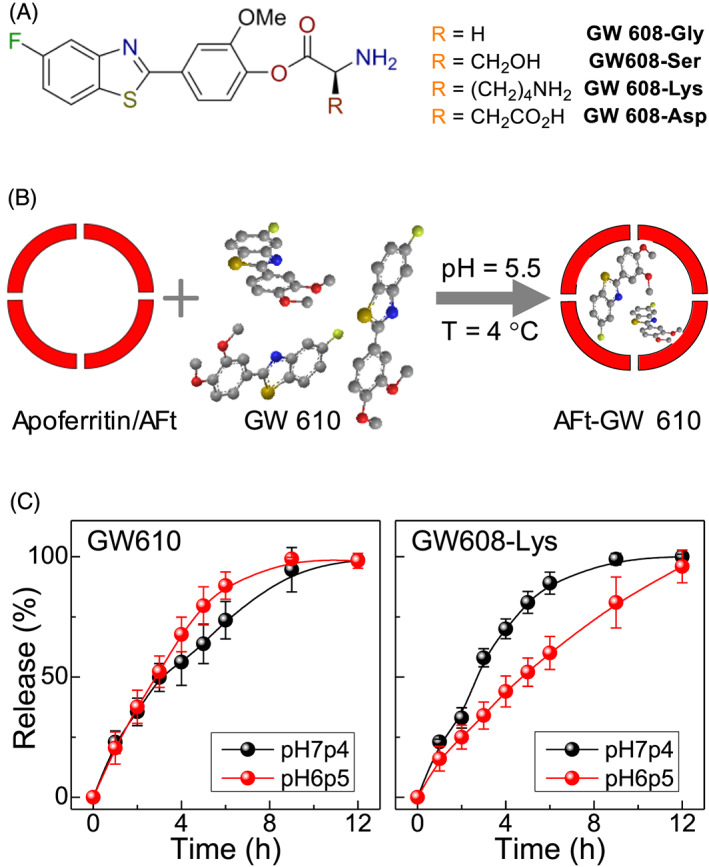
A, Chemical structure of synthesized benzothiazole derivatives. B, Schematic representation of diffusion driven encapsulation of GW 610 into AFt. C, Release profiles of GW 610 and GW 608‐Lys from AFt cavity at 37°C and pH 6.5 (red circles) and 7.4 (black circles)

GW 610 and its derivatives were synthesized following a modified procedure developed by Mortimer et al^23^ (see Supporting Information S1). The common intermediate, 6,6′disulphide *bis*(3‐fluroaniline) was obtained via a three‐step reaction. Briefly, 3‐fluoroaniline was refluxed in acetone with benzoyl chloride and ammonium thiocyanate to yield 1‐(3‐fluorophenyl) thiourea, followed by bromination ring closure to give 2‐amino‐5‐fluorobenzothiazole. This was then converted by hydrolysis with aqueous potassium hydroxide to give the common intermediate. The disulphide was refluxed with the corresponding benzaldehyde and triphenylphosphine with catalytic amounts of *p‐*toluenesulphonic acid. GW 608 amino acid esters (Figure 1A) were produced by coupling GW 608 with the corresponding Boc or Boc/^t^Bu protected amino acid, via a *N,N′*‐dicyclohexylcarbodiimide (DCC) ester coupling under dichloromethane reflux. The protecting groups were removed by 4M HCl in dioxane, to give the GW 608 amino acid esters.

All agents were encapsulated into AFt cavities using the nanoreactor method (Figure 1B and Supporting Information S2). The test agent was added to AFt solution in aliquots with time interval of 30 minutes over a period of 5 hours to avoid protein precipitation and to increase the encapsulation efficiency. Because of the hydrophobic nature of benzothiazoles, the preferred uptake route is likely to be via passive diffusion through the hydrophobic channels in the AFt shell.^10^ The encapsulation was assessed by UV‐vis absorption and confirmed with ^19^F‐NMR (see Supporting Information S2). We achieved encapsulation of approximately 190 molecules of GW 610 and approximately 110 molecules of GW 608 per AFt cage. We ascribe the observed difference to the increased polarity of GW 608, which hinders encapsulation into AFt with a negatively charged cavity.^24^ We note, that encapsulation following one‐step addition of the full quantity of test agent reported in the literature^25^, ^26^ resulted in reduced drug loading values, comparable to those published.

For GW 608 derivatives, we also observe some differences in encapsulation efficiency, with approximately 310 GW 608‐Gly and approximately 206 GW 608‐Ser molecules per AFt cage. With the encapsulation procedure carried out at approximately 7.4 pH and the pK_a_ of the amino groups approximately 9, the overall charge on both agents is likely to be +1. It suggests that increased polarity negatively impacts encapsulation, when comparing related structures (GW 610 vs GW 608 and GW 608‐Gly vs GW 608‐Ser). However, charge also affects the encapsulation, with 180 and 380 molecules per AFt capsule of GW 608‐Asp and GW 608‐Lys, respectively. The approximate pKa values of the Asp and Lys side chains are 4 and 10 respectively, so the overall charges on GW 608‐Asp and GW 608‐Lys are likely to be 0 and +2. Hence, GW 608‐Lys interacts with the negatively charged AFt interface, leading to enhanced encapsulation. Our step‐wise encapsulation strategy and modification of charge and polarity of the agent enabled us to achieve approximately two orders of magnitude greater encapsulation efficiency (386 molecules) per AFt cage compared with that reported for gefitinib,^26^ doxorubicin,^8^, ^25^ or cisplatin.^27^ Improved doxorubicin encapsulation via the reassembly route was recently reported for fusion protein HFt‐PAS, achieving 90 molecules per cage^8^; hence, our encapsulation method offers an advantageous approach for enhanced drug loading.

The poor aqueous solubility of GW 610 was evident from low drug loading of approximately 11%. Improved solubility of the amino acid conjugates increased drug loading more than twofold, with highest value of 25% for the Lys‐conjugate. These results confirm that conjugation with amino acids increases encapsulation efficiency due to favourable polarity and enhanced aqueous solubility.

All encapsulated test agents were stable over a period of at least 3 months with respect to drug loading when stored at *T* = 4°C, as quantified by UV‐vis spectroscopy. For samples stored at room temperature, drug release of up to approximately 50% was observed after 7 days storage. At physiologically relevant temperature (*T* approximately 37°C), all test agents were released from the AFt cage with 100% release observed within 12 hours (Figure 1C). Considering the structural sensitivity of AFt to pH,^13^ faster release may be anticipated at acidic pH 6.5 associated with tumour micro‐environments. However, negligible effect was observed for GW 610 release; indeed, more rapid release of GW 608‐Lys at pH 7.4 compared with pH 6.5 was encountered. We postulate that as pH decreases, the proportion of unionized species on Lys reduces, resulting in greater interaction with the negatively charged AFt interior, impeding GW 608‐Lys release. Hence, steady release of benzothiazoles occurs over 12 hours at physiologically relevant pH and is controlled by electrostatic interactions between the agent and the AFt.

### 2.2 In vitro assessment of therapeutic activity

**Figure 2:**
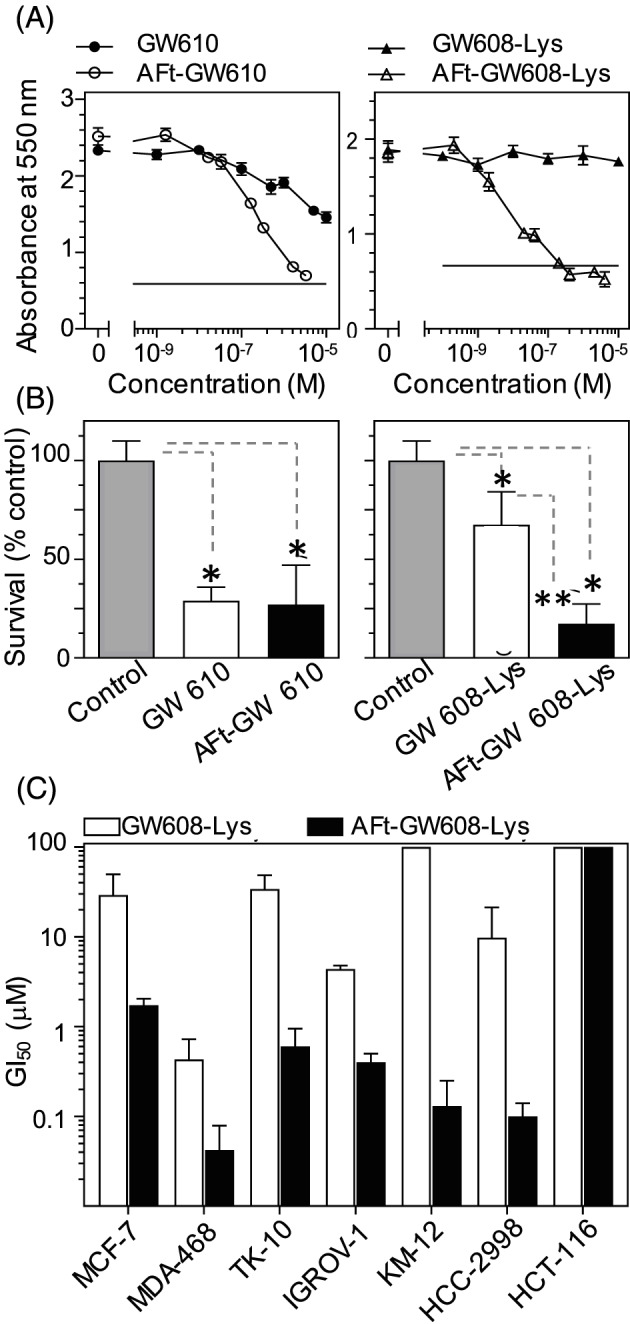
A, Representative dose response curves for KM‐12 cells after 72‐h exposure to GW 610 vs AFt‐GW 610 and GW 608‐Lys vs AFt‐GW 608‐Lys. B, Survival fractions of colonies estimated as a percent of untreated control after 24‐h exposure to GI50 concentration of the agent followed by 7‐day growth. C, Summary of GI50 values for representative cell lines for GW 608‐Lys vs AFt‐GW 608‐Lys. The results are mean ± standard deviation (SD) for n = 4 in one representative trial; number of independent trials is 3; *P < 0.01

**Figure 3:**
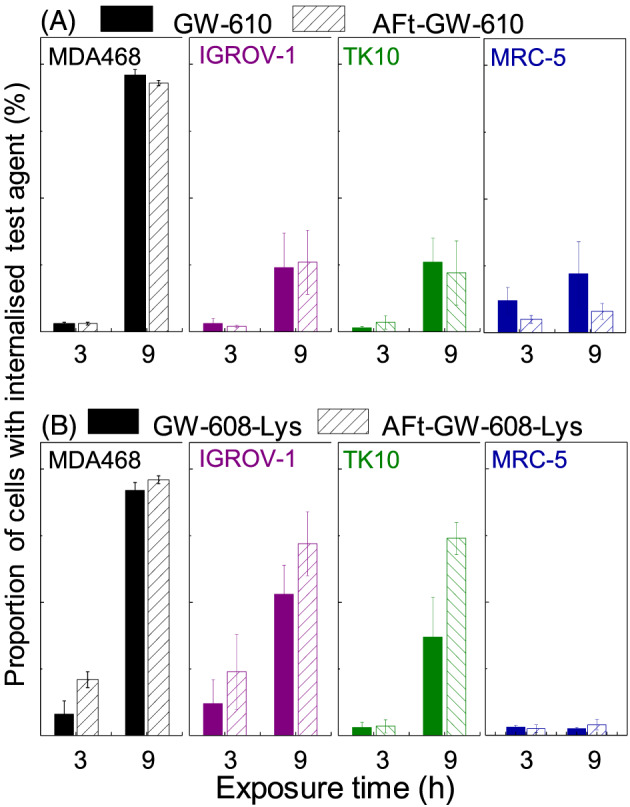
Proportion of cells with intracellular agent in MDA‐468, IGROV‐1, and TK10 carcinoma cell lines and MRC‐5 fibroblasts determined as number of cells with detectable fluorescence of the agent. Cells treated with 1μM concentration of test agent for 3 and 9 h. Results are shown for test agents A, GW 610 and AFt‐GW 610 and B, GW 608‐Lys and AFt‐GW 608‐Lys. Data points are mean ± standard deviation (SD), from three independent trials. For sensitive carcinoma cell lines significant enhancement on uptake is observed for Lys‐conjugated agent compared with insensitive MRC‐5 cells (*P* < 0.001; exemplified also in Figure S7)

MTT in vitro growth inhibitory assays were performed to probe the effects of amino acid conjugation and AFt‐encapsulation on anticancer activity of GW 610. The cell line panel included breast, renal, ovarian, and CRC cell lines. Following 72‐hour exposure, AFt‐GW 610 elicits dose‐dependent growth inhibition against the same cells lines as GW 610 alone; thus, selectivity is retained (Figure 2, Table 1 **,** and Supporting Information S3). AFt‐encapsulated GW 610 is more potent than naked agent in 5/7 cell lines; remarkably, approximately 50‐fold enhanced potency against KM‐12 was observed. In MCF‐7 and MDA‐468, naked GW 610 was more potent than AFt‐encapsulated GW 610, possibly a consequence of abundant cytosolic AhR, which following intracellular diffusion of lipophilic GW 610, results in its rapid sequestration by MCF‐7 and MDA‐468 cells^28^ and subsequent rapid AhR signal transduction cascade activation. Correlating with greater potency of naked GW 610 cf AFt‐GW 610, the proportion of cells with internalized naked GW 610 was greater than that of AFt‐GW 610 in MCF‐7 and MDA‐468 cells (Figure 3A and Supporting Information S3).

**Table 1:** Summary of selected GI_50_ values taken from MTT (72‐h exposure) assay^a^

Test Agent	Mean GI_50_ values ± SD, μM							
	MCF‐7	MDA‐468	TK10	IGROV‐1	KM‐12	HCC‐2998	HCT‐116	MRC‐5
GW 610	0.006 ± 0.004	0.034 ± 0.026	0.57 ± 0.16	3.37 ± 1.68	22.0 ± 6.03	0.32 ± 0.26	27.1 ± 14.3	>100
AFt‐GW 610	0.39 ± 0.33	0.12 ± 0.07	0.14 ± 0.10	0.48 ± 0.44	0.45 ± 0.34	0.11 ± 0.03	2.29 ± 0.67	>100
GW 608‐Lys	29.2 ± 20.6	0.43 ± 0.29	34.1 ± 14.5	4.36 ± 0.41	>100	9.75 ± 11.5	>100	>100
AFt‐GW 608‐Lys	1.72 ± 0.32	0.042 ± 0.037	0.60 ± 0.35	0.40 ± 0.10	0.13 ± 0.12	0.10 ± 0.04	>4.10	>100

a GI_50_ is mean ± standard deviation (SD) from the three trials, where n = 4 per trial.

More polar GW 608 was significantly less potent than GW 610 in all cell lines tested (Table S2) and completely inactive in some cell lines. Reduced lipophilicity of GW 608 is likely to impede diffusion across cell membranes and, combined with potentially lower affinity for AhR, impact negatively on intracellular retention and AhR signal transduction activation.

Conjugation to amino acids increased the potency of GW 608 in all GW 610‐sensitive carcinoma cell lines studied (Table 1 and Supporting Information S3).

Consistent with the selective nature of benzothiazole anticancer activity, negligible activity was observed in insensitive HCT‐116 CRC cells and nontransformed MRC‐5 fibroblasts (GI_50_ > 100μM) (Table 1 and Supporting Information S3), which express neither inducible nor constitutive CYP 1A1 or CYP 2W1. Following encapsulation, the potency of amino acid conjugates was further enhanced compared with the naked agents; furthermore, the selectivity of GW 610 was retained. The AFt‐GW 608‐Gly and AFt‐GW 608‐Ser are both more active than AFt‐GW 610 in MDA‐468 and KM‐12, respectively. AFt‐GW 608‐Lys demonstrated greater activity than AFt‐GW 610 in 4/7 cell lines: MDA‐468, IGROV‐1, KM‐12, and HCC‐2998. Remarkably, following AFt‐encapsulation of GW 608‐Lys, potency enhanced approximately 1000‐fold in KM‐12 cells, and a mean GI_50_ value of 0.13μM was achieved (Figure 2).

Potency of AFt‐GW 608‐Lys cf GW 608‐Lys in sensitive cell lines (Figure 2C) generally correlates with cellular uptake. Cellular uptake of GW 610/GW 608‐Lys was assessed by flow cytometry. We exploited the fluorescent chromophore of the benzothiazole molecule, quantifying the number of cells within the population with detectable fluorescence.^29^ Cells, seeded at the same density, were treated with benzothiazole test agent at a final concentration of 1μM. Analysis revealed that in benzothiazole‐insensitive lines (HCT 116 and MRC‐5), very low numbers of cells retained intracellular GW 610 or GW 608‐Lys following treatment with naked or AFt‐encapsulated agent (eg, less than 8% MRC‐5 fibroblasts tested positive for intracellular GW 610 or GW 608‐Lys). In contrast, a greater proportion of GW 610‐sensitive carcinoma cells carry sequestered GW 610 or GW 608‐Lys (Figure 3 and Supporting Information S3). The proportion of GW 608‐Lys positive MCF‐7, TK10, IGROV‐1, and HCC2998 carcinoma cells was greater following their exposure to AFt‐encapsulated versus naked agent (correlating with greater potency of AFt‐GW 608‐Lys vs naked GW 608‐Lys; Figure 2). Of note also is the stark increase in benzothiazole +ve cells (exemplified by MDA‐MB‐468) between 3‐ and 9‐hour exposure, compared with a lower intracellular accumulation of benzothiazoles encountered in, eg, TK10 cells; these observations are consistent with the slower depletion of antitumour benzothiazoes from nutrient medium of TK10 cells and slower induction of CYP 1A1 expression in these cells. Thus, multiple factors impact the observed population of cells with detectable intracellular fluorescence including: (1) molecular polarity and diffusion across cell membranes; (2) release rates of test agent from AFt cage; (3) the rate of escape from endosomes; (4) cellular sequestration of benzothiazole, dependent upon AhR signal transduction and CYP‐induction/expression and subsequent benzothiazole bioactivation; and (5) fluorescence changes following benzothiazole metabolism. Flow cytometry provides a guide for understanding of the agent uptake in large cell populations. However, to more comprehensively understand uptake, release, and retention, further studies on individual cells would be beneficial, adopting complementary techniques, such as labelling the AFt cage and imaging both delivery vehicle and agent using time‐dependent confocal microscopy.

An additional but distinct test was adopted to thoroughly explore in vitro antitumour activity of AFt‐encapsulated GW 610 formulations. In MCF‐7 clonogenic assays, AFt‐GW 610 (at GI_50_ value) abolished colony formation. Nanomolar concentrations of GW 610 (450nM) and GW 608‐Lys (130nM) were able to significantly reduce KM‐12 colony formation when encapsulated within AFt. Clonogenic assays corroborated in vitro antitumour activity of AFt‐encapsulated GW 610 and amino acid prodrugs. More specifically, they indicate that these formulations possess cytotoxic properties and inhibit formation of progeny colonies.

## 3 CONCLUSIONS

In conclusion, GW 610 has been successfully encapsulated within AFt with more than 190 molecules per AFt cage, and the encapsulated agent is more potent than naked GW 610 in 5/7 cell lines tested, demonstrating up to 50‐fold enhanced activity against KM‐12 cells. Conjugation to amino acids improved solubility and increased encapsulation efficiency of the agent. The most promising of these was the GW 608‐Lys conjugate whose potency was significantly increased following AFt‐encapsulation. The AFt cage enables sustained steady release of the agent over 12 hour at physiological conditions (pH and temperature). Cellular uptake of AFt‐encapsulated and naked agents showed that AFt‐GW 608‐Lys is generally more readily and rapidly sequestered by cells, correlating with more potent growth inhibitory properties. We conclude that AFt‐GW 608‐Lys, which combines potent and selective antitumour activity of parent GW 610 with biocompatibility of AFt delivery vehicle, presents a viable putative anticancer therapy worthy of further preclinical development.

## 4 METHODS

All commercially available starting materials were used without further purification. Nuclear magnetic resonance (NMR) spectra were recorded on a Bruker AV (II) 500 at 500 MHz. High resolution mass spectra (HRMS) were recorded on a Bruker microOTOFII with electrospray ionization (ESI).

### 4.1 AFt encapsulation and drug release studies

AFt was prepared from horse spleen Ft (Sigma Aldrich) following a protocol developed Wong et al^30^ (see Supporting Information S2). We have shown in previous work encapsulating colloidal quantum dots in human H‐ and horse spleen AFt, that the encapsulation methodology developed for one AFt type can be directly translated to the other AFt type.

For encapsulation at 4°C, AFt (pH 7.4, 1.0 mL, 3.2 mg mL^−1^, 7.3 × 10^−9^ mol) was diluted ×2 in Hepes buffer (20mM, pH 7.4); test agent (10mM in DMSO, 0.73 μL, 7.3 × 10^−7^ mol) was added 10 times at 30‐minute intervals under stirring. The resulting solution was dialyzed in tubing MWCO 12‐1400 against Hepes buffer (20mM, pH 7.4, 1.5 L) for 16 hours to remove unencapsulated test agent, centrifuged at 2.4 *g* for 5 minutes at 4°C to remove AFt precipitate, and the supernatant was stored at 4°C.

The protein concentration of AFt was determined by Bradford Assay. The test agent concentrations were determined by UV‐vis absorbance on Varian Cary50 spectrometer. Encapsulation efficiency (EE) and drug loading (DL) were calculated as amount of loaded drug vs amount of total drug added and vs total amount of AFt/drug composite, respectively.

Samples of AFt‐GW 610 and AFt‐GW 608‐Lys (2 mL) (0.2‐0.4 mg mL^−1^ AFt concentration) were placed into dialysis bags (MWCO 3.5 kDa) and immersed in 1.5 L of phosphate buffered saline (PBS) at pH 6.5 and 7.4 (under N_2_ at 37°C). PBS was refreshed every 12 h. Aliquots (10 μL) from the dialysis bag were analysed by UV‐vis absorbance.

### 4.2 In vitro cell culture studies

All cell lines were purchased from The American Type Culture Collection (ATCC) and cultured in RPMI 1640 medium supplemented with 10% foetal bovine serum (FBS) at 37°C in an atmosphere of 5% CO_2_. Cells were passaged twice weekly to maintain continuous logarithmic growth.

For MTT assays, cells were seeded at a density of 5 × 10^3^ cells per well into 96‐well plates and allowed 24 hours to adhere. Serial dilutions were prepared in medium and added to cells (n = 4 per concentration). Viable cells at the time of test agent addition (T0) and following 72 hours of drug exposure were determined by cell‐mediated 3‐(4,5‐dimethylthiazol‐2‐yl)‐2,5‐diphenyltetrazolium bromide (MTT) reduction. MTT was added to each well (final concentration 400 μg mL^−1^) and incubated for 3 hours at 37°C. Well supernatants were aspirated and formazan solubilized with 150 μL DMSO. Absorbance was read at 550 nm using Perkin Elmer Envision plate reader. The GI_50_ values were determined by interpolation.

For cellular uptake studies, cells were seeded at a density of 2.5 × 10^5^ cells per well into 12‐well plates and allowed 24 hours to adhere. Cells were exposed to test agent (1 μM) for 3, 6 or 9 hours before being washed, harvested and fixed in 3.7% formaldehyde. Analyses were performed on a Beckman Coulter MoFlo Astrios EQ, equipped with 355‐nm UV laser, 448/59 detection filter, and Summit 5.1 software. For colonogenic assays, 250 cells per well were seeded into six‐well plates and allowed 24 hours to attach. Test compounds were added at GI_50_ concentrations. After 24‐hour exposure, the medium was aspirated and cells washed with PBS (2 × 1 mL) before incubation in medium (2 mL) for 7 to 9 days. Experiments were terminated when colonies more than or equal to 50 cells were observed in control wells. Colonies were washed (PBS, 2 × 1 mL), fixed (methanol, 15 minutes), stained (0.5% methylene blue, 15 minutes), and counted.

### 4.3 Data analysis

All experiments were repeated at least three times and results are reported as mean ± standard deviation (SD).

## CONFLICT OF INTEREST

Authors have no conflict of interest to declare.

## AUTHORS' CONTRIBUTIONS

All authors had full access to the data in the study and take responsibility for the integrity of the data and the accuracy of the data analysis. *Conceptualization*, G.W., T.D.B., L.T.; *Methodology*, A.F.B., G.W., T.D.B., L.T.; *Investigation*, A.F.B.; *Formal Analysis*, A.F.B., G.W., T.D.B., L.T.; *Resources*, G.W., T.D.B.; *Writing ‐ Original Draft*, A.F.B.; *Writing ‐ Review & Editing*, G.W., T.D.B., L.T.; *Visualization*, A.F.B., L.T.; *Supervision*, G. W., T.D.B., L.T.; *Funding Acquisition*, G.W., T.D.B.

## Supporting information


**Figure S1.** Reagents: (i) Benzoyl chloride, ammonium thiocyanate, acetone, reflux, 1 h; (ii) Br2, CH2Cl2, reflux, 3 h; (iii) KOH(aq), H2O, reflux, 24 h; (iv) PPh3, p‐TsOH, toluene, reflux; (v) Boc / tBu‐amino acid, Et3N, HOBt, DCC, CH2Cl2, 24 h; (vi) 4 M HCl in dioxane, 2‐16 h.
**Figure S2 (a)** A representative UV‐vis absorption spectra for encapsulated GW 610 and GW 610‐Lys (10 μM). **b)** a 19F NMR spectrum of encapsulated GW 610 (100 μM).
**Table S1**: Summary of results of encapsulation.
**Figure S3**. MTT assay (72hr) dose response curves of GW 610 & AFt‐GW 610 in A) MCF‐7, B) MDA‐468, C) TK‐10, D) IGROV‐1, E) KM‐12, F) HCC‐2998 G) HCT‐116 and H) MRC‐5 cells. Data points are mean ± SD, n = 4 in one representative trial, number of independent trials= 3. Inset: Clonogenic assay displaying Survival fractions following 24 exposure treated at GI50 values in corresponding cell lines. Data points are mean ± SD, taken from three independent trials where *n* = 3.
**Figure S4**. MTT assay (72hr) MTT assay (72hr) dose response curves of GW 608‐Lys & AFt‐GW 608‐Lys in A) MCF‐7, B) MDA‐468, C) TK‐10, D) IGROV‐1, E) KM‐12, F) HCC‐2998, G) HCT‐116 and H) MCR‐5 cells. Data points are mean ± SD, n = 4 in one representative trial, number of independent trials = 3. Inset: Clonogenic assay displaying Survival fractions following 24 exposure treated at GI50 values in corresponding cell lines. Data points are mean ± SD, taken from three independent trials where *n* = 3 per trial.
**Table S2:** Summary of GI50 values taken from MTT (72 h exposure) assay. GI50 is mean ± SD from the three trials, where *n* = 4 per trial.
**Figure S5:** Cellular uptake of GW 610 and AFt‐GW 610 A) MCF‐7, B) MDA‐368, C) TK‐10, D) IGROV‐1, E) HCT‐116 and F) MRC‐5 cells, over 3, 6 and 9 hr exposure. Data points are mean ± SD, from three independent trials.
**Figure S6:** Cellular uptake of GW 608‐Lys and AFt‐GW 608‐Lys in A) MCF‐7, B) MDA‐368, C) TK‐10, D) IGROV‐1, E) KM‐12, F) HCC‐2998 G) HCT‐116 and H) MRC‐5 cells, over 3, 6 and 9 hr exposure. Data points are mean ± SD, from three independent trials.
**Figure S7:** Comparison of cellular uptake of AFt encapsulated GW 610 and GW 608‐Lys in representative cell lines following 3h, 6h and
