# Supplementary material for: Development of novel apoferritin formulations for antitumour benzothiazoles
Source: Cancer Rep (Hoboken). 2019 Jan 16;2(4):e1155. doi: 10.1002/cnr2.1155 (PMC7941424; doi:10.1002/cnr2.1155)
Supplement: Supplementary file 1 — Figure S1. Reagents: (i) Benzoyl chloride, ammonium thiocyanate, acetone, reflux, 1 h; (ii) Br2, CH2Cl2, reflux, 3 h; (iii) KOH(aq), H2O, reflux, 24 h; (iv) PPh3, p‐TsOH, toluene, reflux; (v) Boc / tBu‐amino acid, Et3N, HOBt, DCC, CH2Cl2, 24 h; (vi) 4 M HCl in dioxane, 2‐16 h. Figure S2 (a) A representative UV‐vis absorption spectra for encapsulated GW 610 and GW 610‐Lys (10 μM). b) a 19F NMR spectrum of encapsulated GW 610 (100 μM). Table S1: Summary of results of encapsulation. Figure S3. MTT assay (72hr) dose response curves of GW 610 & AFt‐GW 610 in A) MCF‐7, B) MDA‐468, C) TK‐10, D) IGROV‐1, E) KM‐12, F) HCC‐2998 G) HCT‐116 and H) MRC‐5 cells. Data points are mean ± SD, n = 4 in one representative trial, number of independent trials= 3. Inset: Clonogenic assay displaying Survival fractions following 24 exposure treated at GI50 values in corresponding cell lines. Data points are mean ± SD, taken from three independent trials where n = 3. Figure S4. MTT assay (72hr) MTT assay (72hr) dose response curves of GW 608‐Lys & AFt‐GW 608‐Lys in A) MCF‐7, B) MDA‐468, C) TK‐10, D) IGROV‐1, E) KM‐12, F) HCC‐2998, G) HCT‐116 and H) MCR‐5 cells. Data points are mean ± SD, n = 4 in one representative trial, number of independent trials = 3. Inset: Clonogenic assay displaying Survival fractions following 24 exposure treated at GI50 values in corresponding cell lines. Data points are mean ± SD, taken from three independent trials where n = 3 per trial. Table S2: Summary of GI50 values taken from MTT (72 h exposure) assay. GI50 is mean ± SD from the three trials, where n = 4 per trial. Figure S5: Cellular uptake of GW 610 and AFt‐GW 610 A) MCF‐7, B) MDA‐368, C) TK‐10, D) IGROV‐1, E) HCT‐116 and F) MRC‐5 cells, over 3, 6 and 9 hr exposure. Data points are mean ± SD, from three independent trials. Figure S6: Cellular uptake of GW 608‐Lys and AFt‐GW 608‐Lys in A) MCF‐7, B) MDA‐368, C) TK‐10, D) IGROV‐1, E) KM‐12, F) HCC‐2998 G) HCT‐116 and H) MRC‐5 cells, over 3, 6 and 9 hr exposure [file CNR2-2-e1155-s001.pdf]

Copyright WILEY-VCH Verlag GmbH & Co. KGaA, 69469 Weinheim, Germany, 2016.

Supporting Information

Development of novel apoferritin formulations for antitumour benzothiazoles

Alastair F. Breen, Geoffrey Wells, Lyudmila Turyanska and Tracey D Bradshaw

S1. Synthesis and NMR characterization of GW 610 derivatives

The scheme of the reaction used for synthesis of GW 610 derivatives is shown in Figure SI. The procedures used and NMR studies of all products and described in details below.

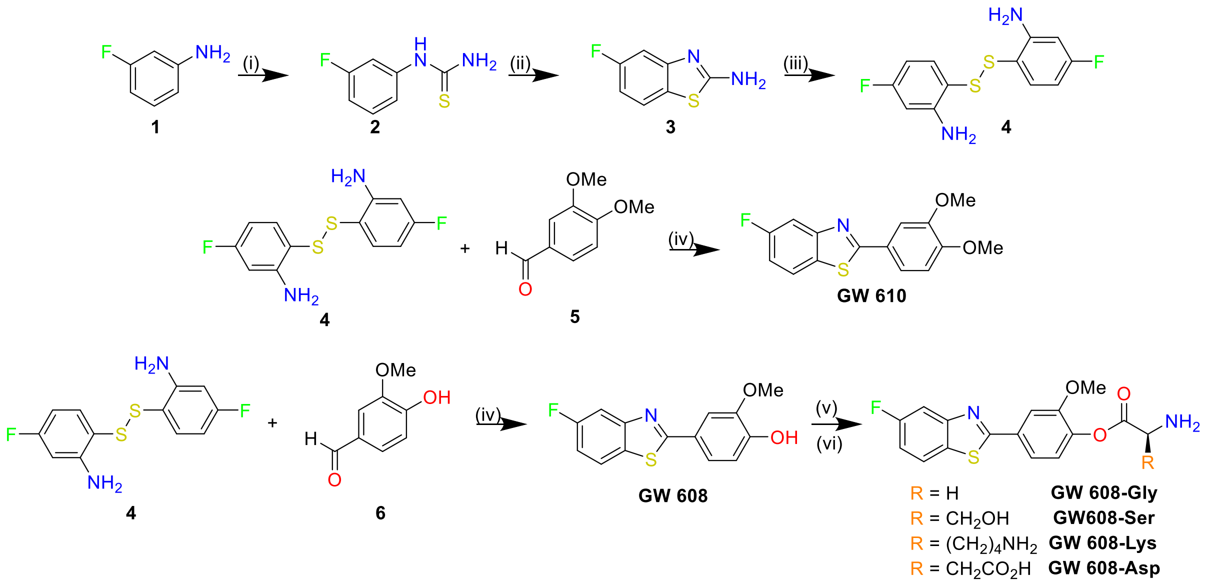

Figure S1. Reagents: (i) Benzoyl chloride, ammonium thiocyanate, acetone, reflux, 1 h; (ii) Br_2, CH_2Cl_2, reflux, 3 h; (iii) KOH(aq), H_2O, reflux, 24 h; (iv) PPh_3, p-TsOH, toluene, reflux; (v) Boc / ^{t}Bu-amino acid, Et_3N, HOBt, DCC, CH_2Cl_2, 24 h; (vi) 4 M HCl in dioxane, 2-16 h.

Synthesis of 1-(3-fluorophenyl)thiourea (2): To a solution of ammonium thiocyanate (2.28 g, 30.00 mmol) in acetone (60 mL) was added benzoyl chloride (3.26 mL, 28.00 mmol). The resultant suspension was refluxed for 1 h, removed from the heat and 3-fluoroaniline (**1**) (1.92 mL, 20.00 mmol) was added over 1 min. The reaction was stirred at reflux for a further 2 h, poured into ice (200 mL), stirred for 15 min, filtered and dried. The solid residue was added to NaOH (10% w/v, 100 mL) and heated at 80 °C for 16 h, cooled and extracted with EtOAc

(3×100 mL). The organic phase was dried over $MgSO_4$, filtered and concentrated under reduced pressure to yield 1-(3-fluorophenyl)thiourea (**2**) as a white solid (3.31 g, 19.5 mmol, 97.5% yield). ^1H NMR (400 MHz, DMSO-d_6) δ 9.88 (s, 1H), 7.55 (dt, J = 11.5, 2.3 Hz, 1H), 7.34 (td, J = 8.2, 6.8 Hz, 1H), 7.17 (ddd, J = 8.1, 2.0, 0.9 Hz, 1H), 6.92 (tdd, J = 8.5, 2.5, 0.9 Hz, 1H). ^{13}C NMR (101 MHz, DMSO-d_6) δ 181.07, 161.84 (d, J = 241.6 Hz), 141.11 (d, J = 10.8 Hz), 130.10 (d, J = 9.6 Hz), 118.14 (d, J = 2.6 Hz), 110.48 (d, J = 21.1 Hz), 109.15 (d, J = 25.3 Hz). HRMS (ESI): calculated for $C_7H_8FN_2S$ $[M+H]^+$ 171.0387, found 171.0394.

Synthesis of 2-amino-5-fluorobenzothiazole (3): To a solution of 1-(3-fluorophenyl)thiourea (**2**) (3.00 g, 18 mmol, 1 eq.) in CH_2Cl_2 (60 mL) was added a solution of bromine (1.05 mL, 20 mmol, 1.1 eq.) in CH_2Cl_2 (12 mL), ensuring the internal temperature remained below 30 °C. The reaction was then refluxed for 16 h, cooled and the precipitate collected. The precipitate was then suspended in H_2O (150 mL), basified with sat. $NaHCO_3$ (aq) and extracted with EtOAc (100 mL). The organic phase was dried over $MgSO_4$, filtered and concentrated *in vacuo* to yield 2-amino-5-fluorobenzothiazole (**3**) as a white solid (2.25 g, 13.4 mmol, 77% yield). ^1H NMR (400 MHz, DMSO-d_6) δ 7.70 – 7.61 (m, 1H), 7.13 (dd, J = 10.5, 2.6 Hz, 0H), 6.85 (td, J = 9.0, 2.6 Hz, 0H). ^{13}C NMR (101 MHz, DMSO-d_6) δ 168.73, 161.23 (d, J = 237.8 Hz), 154.09 (d, J = 12.5 Hz), 126.38 (d, J = 1.9 Hz), 121.62 (d, J = 10.2 Hz), 107.92 (d, J = 23.9 Hz), 104.26 (d, J = 24.2 Hz). HRMS (ESI): calculated for $C_7H_6FN_2S$ $[M+H]^+$ 169.0230, found 169.0231.

Synthesis of 6,6'disulphide *bis*(3-fluroaniline) (4): To a solution of 50% w/v KOH (20 mL) was added 2-amino-5-fluorobenzothiazole (2.0 g, 12 mmol) and the resultant suspension refluxed for 16 h. The solution was cooled, acidified to pH 6 with acetic acid, diluted with H_2O (40 mL) and stirred for 24 h at room temperature. The precipitate was collected and purified by recrystallisation (ethanol/ water) to yield 6,6'disulphide *bis*(3-fluroaniline) (**4**) as a yellow solid (0.976 g, 3.5 mmol, 30% yield). ^1H NMR (400 MHz, DMSO-d_6) δ 6.90 (dd, J = 8.6, 6.8 Hz, 2H), 6.51 (dd, J = 11.7, 2.8 Hz, 2H), 6.23 (td, J = 8.6, 2.8 Hz, 2H), 5.83 (s, 4H). ^{13}C NMR

(101 MHz, DMSO-d_6) δ 164.58 (d, *J* = 244.4 Hz), 151.99 (d, *J* = 12.9 Hz), 138.12 (d, *J* = 11.1 Hz), 111.91 (d, *J* = 2.1 Hz), 102.83 (d, *J* = 22.5 Hz), 100.34 (d, *J* = 25.1 Hz). HRMS (ESI): calculated for $C_{12}H_{11}F_2N_2S_2$ $[M+H]^+$ 285.0326, found 285.0327.

General method A: Synthesis of 2-arylbenzothiazoles substituted on the benzothiazole ring: Disubstituted benzaldehyde (**5**/**6**) (3.5 mmol, 2 eq.), *p*-toulenesulphonic acid (0.35 mmol, 0.2 eq.) and triphenylphosphine (1.75 mmol, 1 eq.) were added to a solution of *bis*(2-amino-4-fluorophenyl) disulphide (**4**) (0.5 g, 1.75 mmol, 1eq.) in toluene (12 mL). The reaction was heated under reflux for 24 h, cooled and concentrated under vacuum. The crude product was purified by column chromatography (petrol ether 40-60 3:2 ethyl acetate) to give the required 2-arylbenzothiazoles. The following compounds were prepared:

5-Fluoro-2-(3,4-dimethoxyphenyl)benzothiazole (GW 610) was formed from 3,4-dimethoxybenzaldehyde, to give a white solid (0.46 g, 1.59 mmol, 91% yield). ^{1}H NMR (400 MHz, DMSO-d_6) δ 8.14 (dd, *J* = 8.8, 5.4 Hz, 1H), 7.86 (dd, *J* = 9.9, 2.5 Hz, 1H), 7.65 – 7.61 (m, 2H), 7.33 (td, *J* = 9.1, 2.6 Hz, 1H), 7.14 (d, *J* = 8.9 Hz, 1H), 3.89 (s, 3H), 3.86 (s, 3H). ^{13}C NMR (101 MHz, DMSO-d_6) δ 170.03, 161.39 (d, *J* = 240.5 Hz), 154.49 (d, *J* = 12.4 Hz), 151.84, 149.12, 125.33, 123.48 (d, *J* = 10.0 Hz), 121.03, 113.45 (d, *J* = 24.7 Hz), 111.96, 109.38, 108.50 (d, *J* = 23.9 Hz), 55.76, 55.64. HRMS (ESI): calculated for $C_{15}H_{12}FNO_2S$ $[M+H]^+$ 290.0689, found 290.0683.

5-Fluoro-2-(4-hydroxy-3-methoxyphenyl)benzothiazole (GW 608) was formed from 4-hydroxy-3-methoxy benzaldehyde, to give a white solid (2.25 g, 8.2 mmol, 40% yield). ^{1}H NMR (400 MHz, DMSO-d_6) δ 9.90 (s, 1H), 8.12 (dd, *J* = 8.8, 5.4 Hz, 1H), 7.83 (dd, *J* = 10.0, 2.5 Hz, 1H), 7.61 (d, *J* = 2.1 Hz, 1H), 7.51 (dd, *J* = 8.2, 2.1 Hz, 1H), 7.31 (td, *J* = 9.0, 2.6 Hz, 1H), 6.94 (d, *J* = 8.2 Hz, 1H), 3.89 (s, 3H). ^{13}C NMR (101 MHz, DMSO-d_6) δ 170.32, 161.36 (d, *J* = 240.7 Hz), 154.48, 150.32, 148.09, 129.95, 124.07, 123.42, 121.39, 115.93, 113.19 (d,

J = 24.8 Hz), 110.04, 108.34 (d, J = 23.9 Hz), 55.70. HRMS (ESI): calculated for $C_{14}H_{11}FNO_2S$ $[M+H]^+$ 276.0489, found 276.0487.

General method B: Synthesis of GW 608 esters from Boc-amino acids: A solution of 5-fluoro-2-(4-hydroxy-3-methoxyphenyl) benzothiazole (GW 608) (0.35 mmol, 1 eq) in CH_2Cl_2 (1.5 mL) was added to a solution the *N*-Boc-amino acid (0.7 mmol, 2eq), triethylamine (0.07 mmol, 0.2 eq) and HOBt (0.035 mmol, 0.1 eq) in CH_2Cl_2 (7.5 mL) and stirred at room temperature. The resulting solution was treated with a solution of *N,N'*-dicyclohexylcarbodiimide (DCC) (1.13 mmol, 1.5 eq) in CH_2CL_2 (1.5 mL) added dropwise. The reaction was stirred at room temperature for 24 h, and then cooled to 0 ºC in an ice bath. The precipitated DCU was removed by filtration and the filtrate washed with Na_2CO_3 solution (2% m/v, 15 mL) and H_2O (20 mL), then concentrated under reduced pressure. The crude product was purified by recrystallization from MeOH/H_2O, to give the required ester. The following compounds were prepared:

GW 608-Gly-Boc was formed from Boc-Gly-OH, to give a white powder (0.122 g, 28 mmol, 80% yield). ^1H NMR (400 MHz, DMSO-d_6) δ 8.21 (dd, J = 8.9, 5.4 Hz, 1H), 7.94 (dd, J = 9.9, 2.6 Hz, 1H), 7.79 (d, J = 2.1 Hz, 1H), 7.68 (dd, J = 8.2, 2.0 Hz, 1H), 7.45 – 7.36 (m, 2H), 7.30 (d, J = 8.2 Hz, 1H), 4.01 (d, J = 6.1 Hz, 2H), 3.92 (s, 3H), 1.41 (s, 9H). ^{13}C NMR (101 MHz, DMSO-d_6) δ 169.24, 168.63, 155.82, 151.39, 141.72, 131.54, 123.84, 120.32, 110.76, 78.47, 56.14, 41.80, 28.14. HRMS (ESI): calculated for $C_{21}H_{22}FN_2O_5S$ $[M+H]^+$ 433.1228, found 433.1234.

GW 608-Ser(^{t}Bu)-Boc was formed from Boc-Ser(^{t}Bu)-OH, to give a white solid (152 mg, 0.29 mmol, 84% yield). ^1H NMR (400 MHz, Methanol-d_4) δ 8.01 (dd, J = 8.9, 5.1 Hz, 1H), 7.86 (s, 1H), 7.74 (dd, J = 9.6, 2.5 Hz, 1H), 7.67 (d, J = 8.2 Hz, 1H), 7.32 – 7.20 (m, 2H), 4.59 (s, 1H), 3.96 (s, 3H), 3.79 (dd, J = 9.3, 4.1 Hz, 1H), 1.49 (s, 9H), 1.26 (s, 9H). ^{13}C NMR (101 MHz, Methanol-d_4) δ 153.25, 124.65, 124.22, 112.12, 109.58, 80.96, 63.01, 56.68, 55.99, 28.20 (d, J = 99.0 Hz).). HRMS (ESI): calculated for $C_{26}H_{32}FN_2O_6S$ $[M+H]^+$ 519.1960, found 519.1956.

GW 608-Lys(Boc)-Boc was formed from Boc-Lys(Boc)-OH, to give a white solid (0.13 g, 0.22 mmol, 63% yield). ^{1}H NMR (400 MHz, DMSO-d_6) δ 8.21 (dd, *J* = 8.9, 5.4 Hz, 1H), 7.94 (dd, *J* = 9.8, 2.6 Hz, 1H), 7.79 (d, *J* = 2.0 Hz, 1H), 7.68 (dd, *J* = 8.2, 2.0 Hz, 1H), 7.49 – 7.36 (m, 2H), 7.24 (d, *J* = 8.2 Hz, 1H), 6.81 (s, 1H), 4.27 – 4.13 (m, 1H), 3.90 (s, 3H), 3.00 – 2.89 (m, 2H), 1.95 – 1.50 (m, 4H), 1.42 (s, 9H), 1.38 (s, 9H), 1.30 – 0.98 (m, 2H). ^{13}C NMR (101 MHz, DMSO-d_6) δ 156.08, 151.89, 111.25, 78.90, 77.85, 56.62, 54.26, 28.71 (d, *J* = 9.9 Hz), 23.19. HRMS (ESI): calculated for $C_{30}H_{39}FN_3O_7S$ $[M+H]^+$ 604.2487, found 604.2465.

GW 608-Asp(^{t}Bu)-Boc was formed from Boc-Asp(^{t}Bu)-OH, to give a white solid (184 mg, 0.34 mmol, 96% yield). ^{1}H NMR (400 MHz, Methanol-d_4) δ 8.01 (dd, *J* = 8.9, 5.1 Hz, 1H), 7.85 (d, *J* = 1.9 Hz, 1H), 7.73 (dd, *J* = 9.5, 2.5 Hz, 1H), 7.67 (dd, *J* = 8.2, 2.0 Hz, 1H), 7.32 – 7.20 (m, 2H), 4.81 (t, *J* = 6.5 Hz, 1H), 3.96 (s, 3H), 2.97 (dd, *J* = 16.2, 5.5 Hz, 1H), 2.81 (dd, *J* = 16.3, 7.8 Hz, 1H), 1.49 (d, *J* = 4.8 Hz, 18H). ^{13}C NMR (101 MHz, methanol-d_4) δ 170.93, 153.16, 124.12, 121.43, 112.03, 109.82, 82.65, 56.71, 51.88, 38.76, 28.51 (d, *J* = 39.8 Hz). HRMS (ESI): calculated for $C_{27}H_{32}FN_2O_7S$ $[M+H]^+$ 547.1909, found 547.1890.

General method C: Deprotection of GW 608-amino acid-Boc esters: GW 608-amino acid-Boc esters (0.1 g) were dissolved in 4 M HCl in dioxane (4 mL) and stirred for 1.5 h at room temperature. The precipitate was collected and washed with dioxane to give the corresponding hydrochloride salt. The following compounds were prepared:

GW 608-Gly was formed from GW 608-Gly-Boc, to give a pale yellow solid (62 mg, 0.19 mmol, 83% yield). ^{1}H NMR (400 MHz, DMSO-d_6) δ 8.61 (d, *J* = 5.7 Hz, 3H), 8.23 (dd, *J* = 8.9, 5.4 Hz, 1H), 7.94 (dd, *J* = 9.8, 2.5 Hz, 1H), 7.84 (d, *J* = 2.0 Hz, 1H), 7.73 (dd, *J* = 8.3, 2.0 Hz, 1H), 7.45 – 7.37 (m, 2H), 4.19 (d, *J* = 5.6 Hz, 2H), 3.95 (s, 3H). ^{13}C NMR (101 MHz, DMSO-d_6) δ 169.08, 166.01, 154.35, 151.19, 140.90, 132.10, 130.54, 123.71, 120.43, 110.94, 108.92 (d, *J* = 23.8 Hz), 56.24. HRMS (ESI): calculated for $C_{16}H_{14}FN_2O_3S$ $[M+H]^+$ 333.0704, found 333.0708.

GW 608-Ser was formed from GW 608-Ser(^{t}Bu)-Boc, to give a white solid. (70 mg, 0.19 mmol, 66% yield). ^{1}H NMR (400 MHz, methanol-d_4) δ 8.03 (dd, *J* = 8.9, 5.1 Hz, 1H), 7.90 (d, *J* = 1.9 Hz, 1H), 7.75 (dd, *J* = 9.5, 2.5 Hz, 1H), 7.71 (dd, *J* = 8.3, 2.0 Hz, 1H), 7.34 (d, *J* = 8.3 Hz, 1H), 7.32 – 7.25 (m, 1H), 4.51 (dd, *J* = 5.1, 3.6 Hz, 1H), 4.22 (dd, *J* = 11.8, 5.1 Hz, 1H), 4.15 (dd, *J* = 11.7, 3.6 Hz, 1H), 3.98 (s, 3H). ^{13}C NMR (101 MHz, Methanol-d_4) δ 171.24, 167.15, 155.94, 152.84, 142.87, 134.04, 131.93, 124.44, 121.48, 115.36 (d, *J* = 25.1 Hz), 112.13, 109.73 (d, *J* = 23.9 Hz), 68.14, 60.83, 56.79, 56.14. HRMS (ESI): calculated for $C_{17}H_{16}FN_2O_4S$ [M+H]$^+$ 363.0809, found 363.0814.

GW 608-Lys was formed from GW 608-Lys(Boc)-Boc, to give a white solid (60 mg, 0.15 mmol, 91% yield). ^{1}H NMR (400 MHz, DMSO-d_6) δ 8.85 (d, *J* = 5.1 Hz, 3H), 8.23 (dd, *J* = 8.9, 5.4 Hz, 1H), 8.03 (s, 3H), 7.94 (dd, *J* = 9.8, 2.5 Hz, 1H), 7.84 (d, *J* = 2.0 Hz, 1H), 7.73 (dd, *J* = 8.3, 2.0 Hz, 1H), 7.42 (dd, *J* = 10.7, 7.4 Hz, 2H), 4.36 (d, *J* = 5.0 Hz, 1H), 3.95 (s, 3H), 2.87 – 2.77 (m, 2H), 2.09 – 1.98 (m, 2H), 1.74 – 1.50 (m, 4H). ^{3}C NMR (101 MHz, DMSO-d_6) δ 169.05, 167.65, 151.09, 140.87, 132.18, 130.55, 123.83 (d, *J* = 16.6 Hz), 120.43, 110.92, 66.35, 56.32, 51.70, 38.29, 29.45, 26.32, 21.14. HRMS (ESI): calculated for $C_{20}H_{23}FN_3O_3S$ [M+H]$^+$ 404.1439, found 404.1444.

GW 608-Asp was formed from GW 608-Asp(^{t}Bu)-Boc, to give a yellow solid. (100 mg, 0.26 mmol, 79% yield). ^{1}H NMR (400 MHz, methanol-d_4) δ 8.03 (dd, *J* = 8.8, 5.1 Hz, 1H), 7.90 (d, *J* = 1.9 Hz, 1H), 7.75 (dd, *J* = 9.5, 2.5 Hz, 1H), 7.71 (dd, *J* = 8.3, 1.9 Hz, 1H), 7.33 – 7.26 (m, 2H), 4.71 (t, *J* = 5.2 Hz, 1H), 3.98 (s, 3H), 3.66 (s, 2H). ^{13}C NMR (101 MHz, methanol-d_4) δ 172.38, 171.20, 152.86, 142.78, 134.09, 124.29 (d, *J* = 16.5 Hz), 121.45, 112.11, 109.62, 68.14, 56.73, 50.59, 34.92. HRMS (ESI): calculated for $C_{18}H_{16}FN_2O_5S$ [M+H]$^+$ 391.0758, found 391.0760.

S2. Apoferritin encapsulation and drug release studies

AFt was prepared as previously reported by Wong et al.[31] Briefly a solution of horse spleen ferritin (85 mg mL^{-1}) was diluted ×10 in sodium acetate buffer (NaOAc, 0.1 M, pH 5.5), placed in a dialysis bag (MWCO 12-1400) and immersed in NaOAc buffer (0.1 M, pH 5.5, 1.5 L). The buffer was purged with N_2 for 15 min before thioglycolic acid (0.03 M, 2.0 mL) was added dropwise, the solution was left under constant N_2 purge, after 2 h, more thioglycolic acid (0.03 M, 1.0 mL) was added and the solution was left for 1 h. This process was carried out with fresh buffer 5-6 times until a colourless protein sample was observed. The AFt was then placed in fresh buffer for 24 h before storing in 1 mL aliquots at -18 °C.

For encapsulation at 4 °C, AFt (pH 7.4, 1.0 mL, 3.2 mg mL^{-1}, 7.3×10^{-9} mols) was diluted ×2 in Hepes buffer (20 mM, pH 7.4), test agent (10 mM in DMSO, 0.73 µL, 7.3×10^{-7} mols) was added ten times at 30 min intervals. The 10-times addition of the drug aliquots gives the ratio of AFt to drug molecules of 1:1000. The resulting solution was dialysed in tubing MWCO 12-1400 against Hepes buffer (20 mM, pH 7.4, 1.5 L) for 16 h to remove un-encapsulated test agent, centrifuged at 2.4g for 5 min at 4 °C to remove AFt precipitate and the supernatant was stored in 1 mL aliquots at 4 °C.

To assess any potential loss of protein during encapsulation, the protein concentration of AFt was determined by Bradford Assay.[32] The test agent concentrations were determined by UV absorbance on a Varian Cary50, UV-Vis Spectrometer, using calibration curves obtained from samples of known test agent concentrations (Figure S2a). We also note that AFt is not optically active in the same wavelength range as test agents. The results of encapsulation were confirmed by ^{19}F-NMR (Figure S2b) studies on 500 MHz Bruker Advance III spectrometer. All experiments were performed in triplicate.

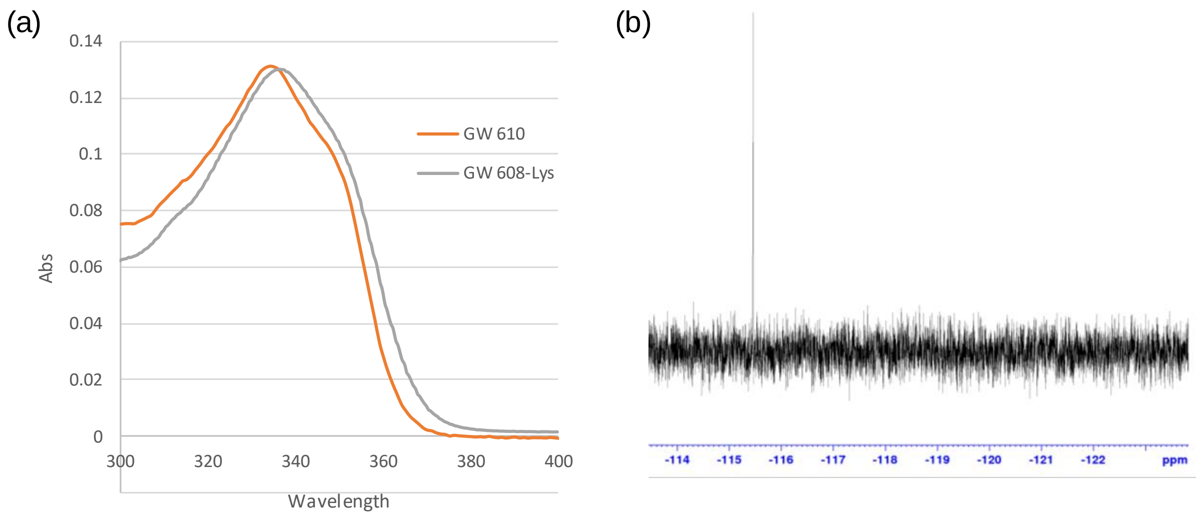

Figure S2 (a) A representative UV-vis absorption spectra for encapsulated GW 610 and GW 610-Lys (10 µM). **b)** a ^{19}F NMR spectrum of encapsulated GW 610 (100 µM).

Encapsulation efficiency (EE) and drug loading (DL) were calculated from Equations S1 and S2 using mass of loaded agent is estimated from UV-vis measurements and actual mass of AFt is estimated from Bradford assay. The results are summarised in the Table S1.

$$EE = \frac{m_{agent}^{loaded}}{m_{agent}^{added}} \times 100\% \qquad \text{(Equation S1)}$$

$$DL = \frac{m_{agent}^{loaded}}{\left(m_{AFt}^{actual} + m_{agent}^{loaded}\right)} \times 100\% \qquad \text{(Equation S2)}$$

Table S1: Summary of results of encapsulation.

	AFt-GW 610	AFt-GW 608	AFt-GW 608-Gly	AFt-GW 608-Ser	AFt-GW 608-Lys	AFt-GW 608-Asp
No of molecules per AFt Cage	191 ± 17	112 ± 25	308 ± 58	206 ± 34	386 ± 34	179 ± 91
Encapsulation efficiency / %	12 ± 6	8 ± 3	13 ± 2	11 ± 2	14 ± 6	9 ± 1
Drug Loading / %	11 ± 1	7 ± 1	15 ± 8	14 ± 2	25 ± 2	13 ± 6

Drug release was studied in phosphate buffered saline (PBS) at pH 6.5 and 7.4. Samples of AFt-GW 610 and AFt-GW 608-Lys (2 mL) (0.2-0.4 mg mL^{-1} AFt concentration) were placed into dialysis bags (MWCO 3.5 kDa) and immersed in 1.5 L PBS at the required pH value. The PBS was purged under N_2 and stirred at 37 °C. To ensure no equilibrium was reached, the PBS was replaced with fresh PBS every 12 h. The amount of test agent released from the AFt was estimated by measuring the amount of drug remaining in the dialysis bag. Aliquots (10 µL) were withdrawn from the dialysis bag and analysed by UV absorbance on a Varian Cary50, UV-Vis Spectrometer, using calibration curves obtained from samples of known test agent concentrations. All experiments were performed in triplicate.

S3. *In vitro* studies

In vitro studies were performed to assess the effect of AFt encapsulation on the potency of the agent. The obtained GI_{50} values or all studies cell lines and test agents are summarized in the Figure S3-S4 and the Table S2.

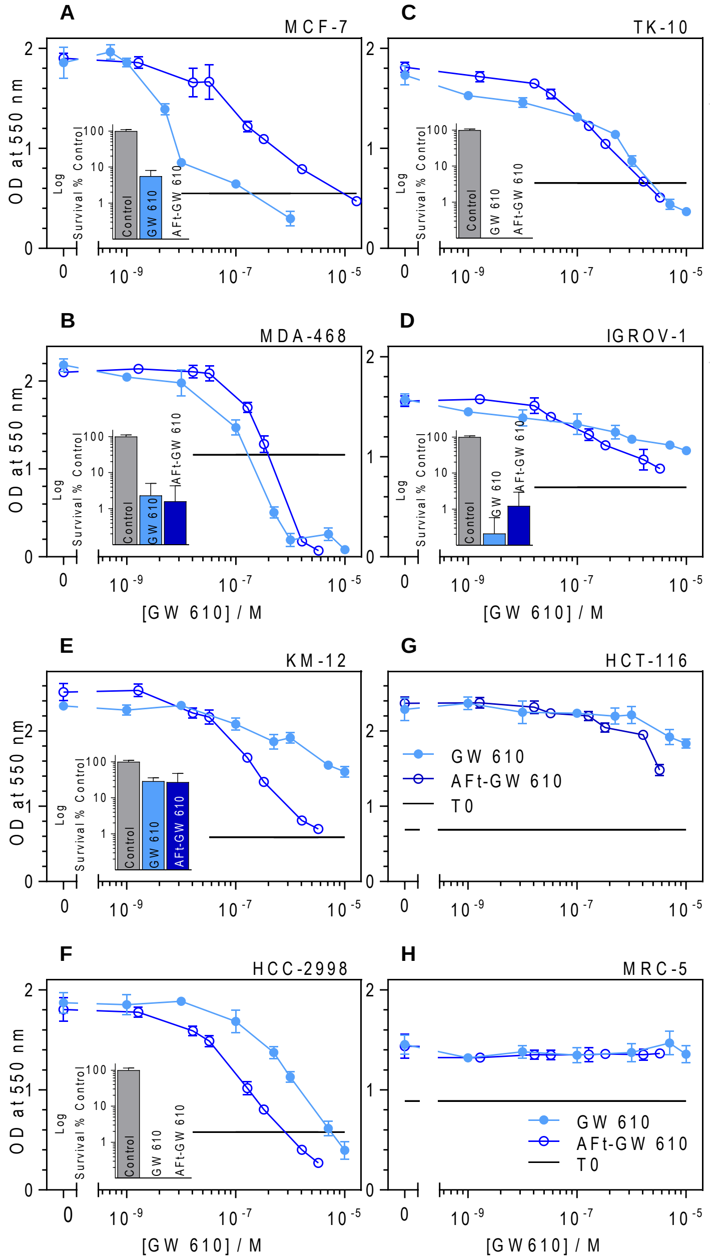

Figure S3. MTT assay (72hr) dose response curves of GW 610 & AFt-GW 610 in A) MCF-7, B) MDA-468, C) TK-10, D) IGROV-1, E) KM-12, F) HCC-2998 G) HCT-116 and H) MRC-5 cells. Data points are mean ± SD, n = 4 in one representative trial, number of independent trials= 3. Inset: Clonogenic assay displaying Survival fractions following 24 exposure treated at GI50 values in corresponding cell lines. Data points are mean ± SD, taken from three independent trials where *n* = 3.

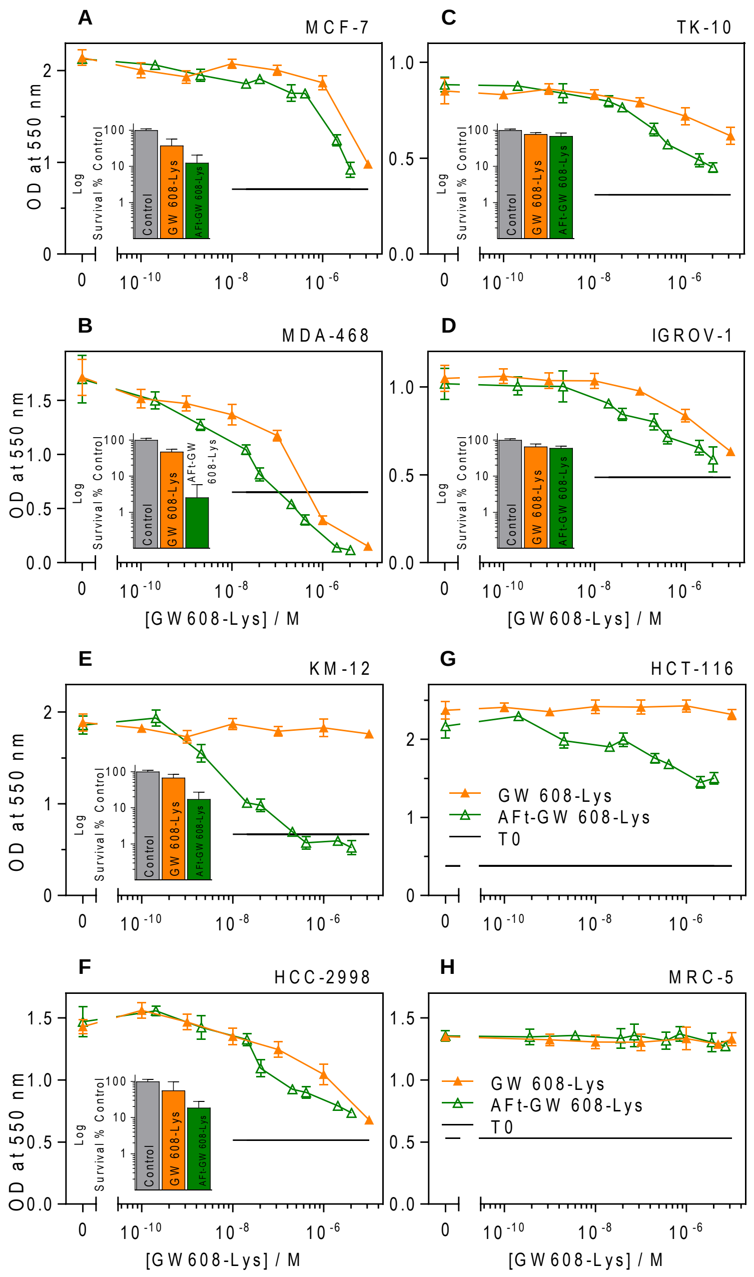

Figure S4. MTT assay (72hr) MTT assay (72hr) dose response curves of GW 608-Lys & AFt-GW 608-Lys in A) MCF-7, B) MDA-468, C) TK-10, D) IGROV-1, E) KM-12, F) HCC-2998, G) HCT-116 and H) MCR-5 cells. Data points are mean ± SD, n = 4 in one representative trial, number of independent trials = 3. Inset: Clonogenic assay displaying Survival fractions following 24 exposure treated at GI50 values in corresponding cell lines. Data points are mean ± SD, taken from three independent trials where *n* = 3 per trial.

Table S2: Summary of GI_{50} values taken from MTT (72 h exposure) assay. GI_{50} is mean ± SD from the three trials, where *n* = 4 per trial.

Test Agent	Mean GI_{50} values ± SD (μM)						
	MCF-7	MDA-468	TK-10	IGROV-1	KM-12	HCC-2998	HCT-116
GW 610	0.006 ± 0.004	0.034 ± 0.026	0.57 ± 0.16	3.37 ± 1.68	22.0 ± 6.03	0.32 ± 0.26	27.1 ± 14.3
AFt-GW 610	0.39 ± 0.33	0.12 ± 0.07	0.14 ± 0.10	0.48 ± 0.44	0.45 ± 0.34	0.11 ± 0.03	2.29 ± 0.67
GW 608	11.2 ± 5.41	0.80 ± 0.62	> 100	8.98 ± 0.23	> 100	6.80 ± 3.31	> 100
AFt-GW608	> 1.33	0.11 ± 0.06	1.07 ± 0.12	0.64 ± 0.02	> 1.33	0.36 ± 0.14	> 1.33
GW608-Gly	11.0 ± 9.74	0.45 ± 0.25	> 100	12.9 ± 6.17	> 100	9.61 ± 6.36	> 100
AFt-GW608-Gly	0.96 ± 1.13	0.032 ± 0.027	1.39 ± 0.15	1.65 ± 1.91	0.60 ± 0.44	0.13 ± 0.05	> 3.43
GW 608-Ser	5.68 ± 2.12	0.75 ± 0.53	74.6 ± 3.37	6.31 ± 2.20	> 100	3.44 ± 1.62	> 100
AFt-GW 608-Ser	2.71 ± 0.11	0.34 ± 0.19	2.61 ± 0.23	1.34 ± 1.77	0.065 ± 0.034	1.39 ± 0.66	> 3.58
GW608-Lys	29.2 ± 20.6	0.43 ± 0.29	34.1 ± 14.5	4.36 ± 0.41	> 100	9.75 ± 11.5	> 100
AFt-GW608-Lys	1.72 ± 0.32	0.042 ± 0.037	0.60 ± 0.35	0.40 ± 0.10	0.13 ± 0.12	0.10 ± 0.04	> 4.10
GW 608-Asp	6.84 ± 2.82	0.64 ± 0.15	> 100	8.53 ± 0.84	> 100	3.31 ± 1.39	> 100
AFt-GW608-Asp	1.86 ± 0.82	0.69 ± 0.11	> 2.80	1.70 ± 0.12	0.92 ± 0.61	1.43 ± 0.22	> 2.80

The results of the MTT assays could be related to the rate of agent uptake by each cell line. Hence the uptake was studied by flow cytometry to quantify the proportion of cells in each cell population with internalized naked agent and was compared to that with internalized AFt encapsulated agent (Figures S5-S6 and Table S3). The concentration of the agent was kept the same (1 μM) for all studies cell lines and test agents.

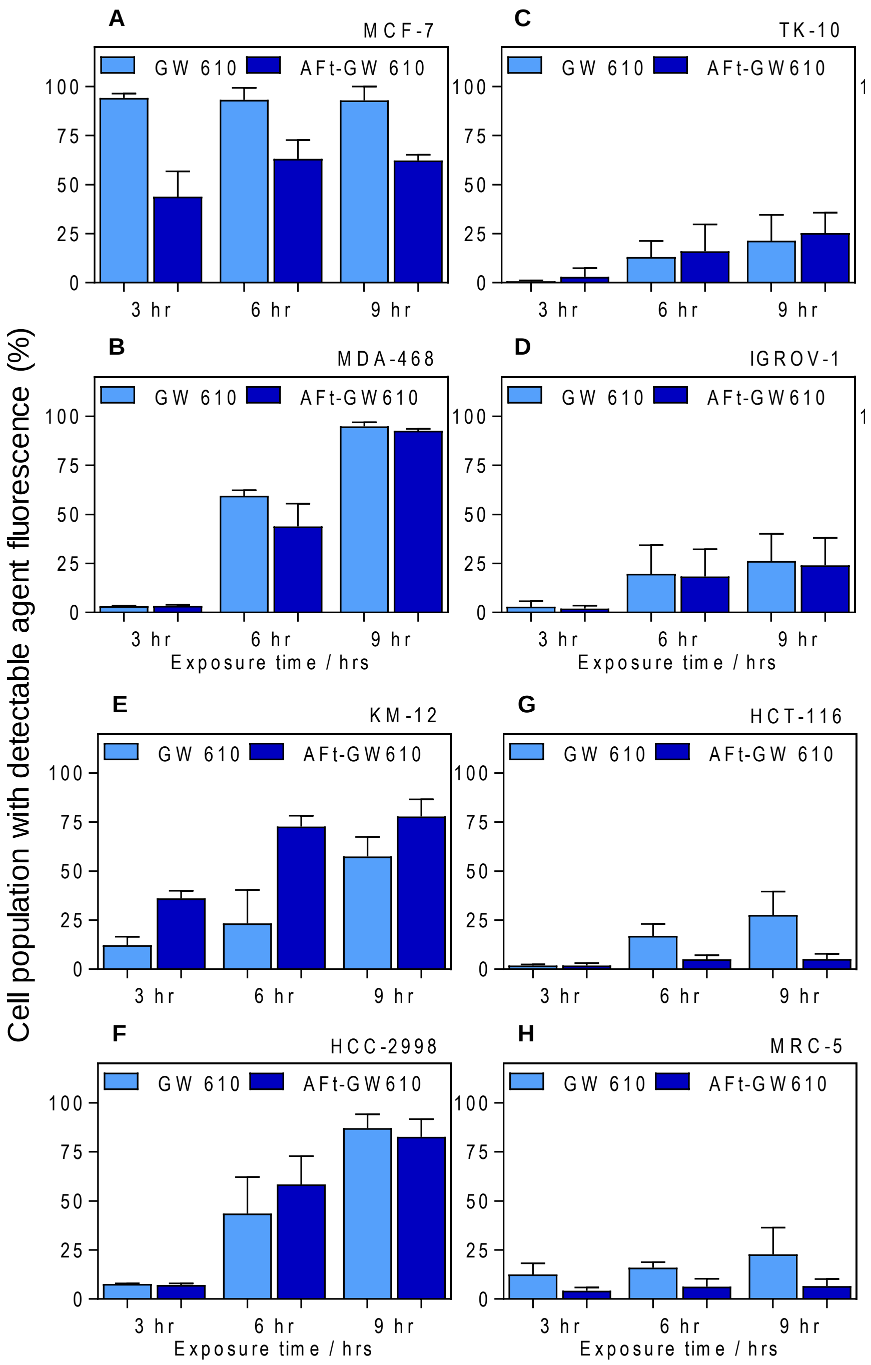

Figure S5: Cellular uptake of GW 610 and AFt-GW 610 A) MCF-7, B) MDA-368, C) TK-10, D) IGROV-1, E) HCT-116 and F) MRC-5 cells, over 3, 6 and 9 hr exposure. Data points are mean ± SD, from three independent trials.

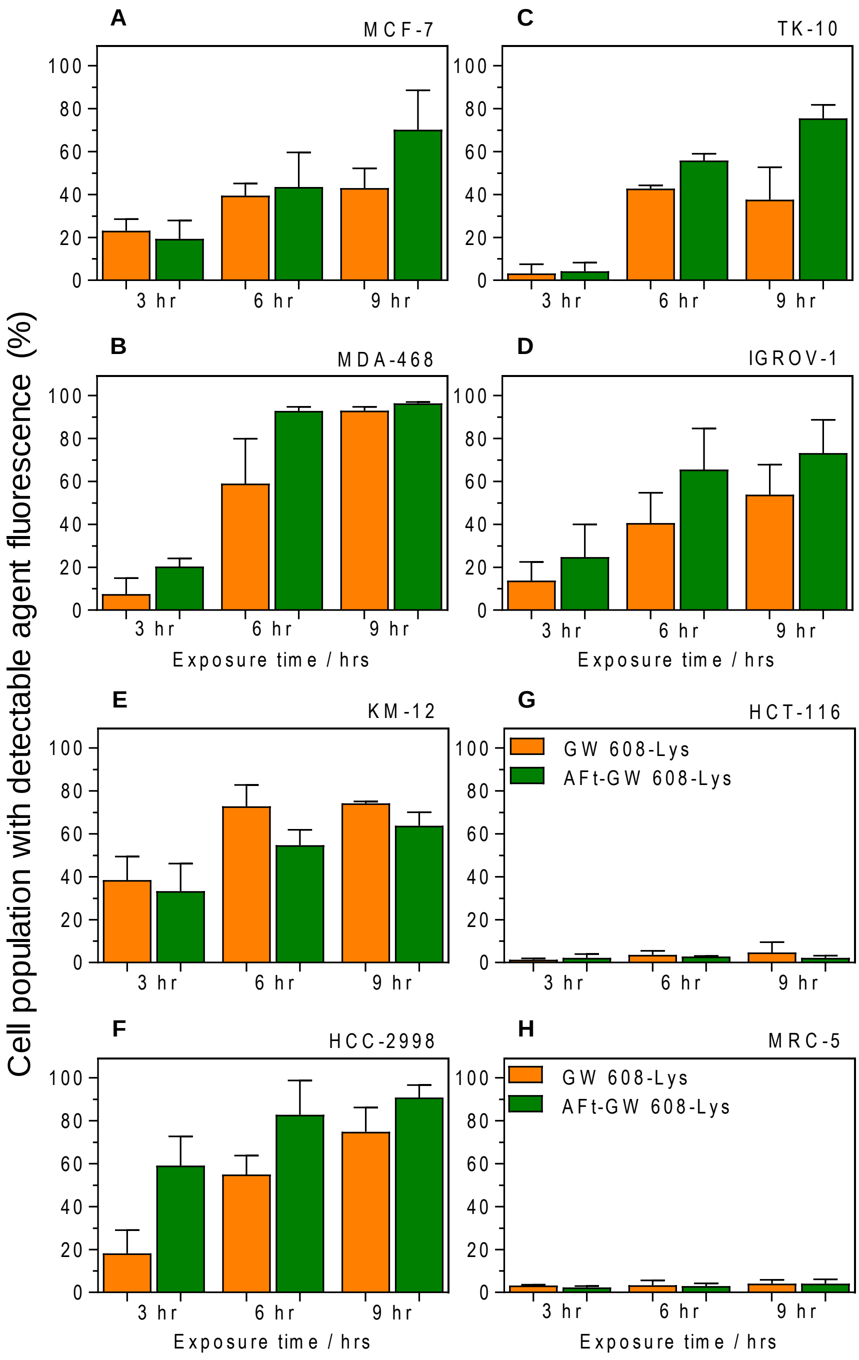

Figure S6: Cellular uptake of GW 608-Lys and AFt-GW 608-Lys in A) MCF-7, B) MDA-368, C) TK-10, D) IGROV-1, E) KM-12, F) HCC-2998 G) HCT-116 and H) MRC-5 cells, over 3, 6 and 9 hr exposure. Data points are mean ± SD, from three independent trials.

Longer retention of agent inside AFt observed for Lys-conjugated agent at reduced pH is benefitial for drug delivery as greater proportion og cells will retain the agent (Figure S7).

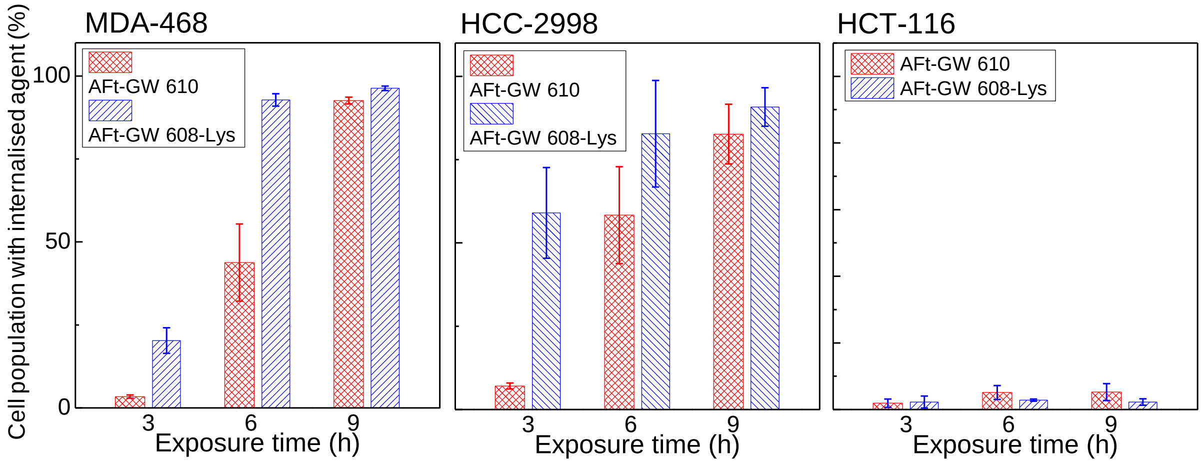

Figure S7: Comparison of cellular uptake of AFt encapsulated GW 610 and GW 608-Lys in representative cell lines following 3h, 6h and
